# New indices to characterize drawing behavior in humans (*Homo sapiens*) and chimpanzees (*Pan troglodytes*)

**DOI:** 10.1038/s41598-021-83043-0

**Published:** 2021-02-16

**Authors:** Lison Martinet, Cédric Sueur, Satoshi Hirata, Jérôme Hosselet, Tetsuro Matsuzawa, Marie Pelé

**Affiliations:** 1 Université de Strasbourg, CNRS, IPHC UMR 7178, Strasbourg, France; 2 Institut Universitaire de France, Paris, France; 3 Kumamoto Sanctuary, Wildlife Research Center, Kyoto University, Kumamoto, Japan; 4 Kyoto University Institute for Advanced Study, Kyoto, Japan; 5 Primate Research Institute, Kyoto University, Kyoto, Japan; 6 ETHICS EA 7446, Lille Catholic University, Lille, Hauts-de-France France

**Keywords:** Biological anthropology, Animal behaviour

## Abstract

Techniques used in cave art suggest that drawing skills emerged long before the oldest known representative human productions (44,000 years bc). This study seeks to improve our knowledge of the evolutionary origins and the ontogenetic development of drawing behavior by studying drawings of humans (N = 178, 3- to 10-year-old children and adults) and chimpanzees (N = 5). Drawings were characterized with an innovative index based on spatial measures which provides the degree of efficiency for the lines that are drawn. Results showed that this index was lowest in chimpanzees, increased and reached its maximum between 5-year-old and 10-year-old children and decreased in adults, whose drawing efficiency was reduced by the addition of details. Drawings of chimpanzees are not random suggesting that their movements are constrained by cognitive or locomotor aspect and we cannot conclude to the absence of representativeness. We also used indices based on colors and time and asked children about what they drew. These indices can be considered relevant tools to improve our understanding of drawing development and evolution in hominids.

## Introduction

“My drawing was not a picture of a hat.

It was a picture of a boa constrictor digesting an elephant.”

Antoine de Saint-Exupéry, *The Little Prince*

Evidence in prehistoric caves and in museums underlines that drawing is one of the most characteristic behaviors of the human species, yet its definition remains vague. Indeed, drawing behavior is considered by some authors as the simple will to mark, to produce visible traces but it can also be etymologically understood as something more complex such as a design, or a goal to reach^1^. This study considers drawing behavior in its simplest form, i.e. an active creation of visible marks that may or may not be figurative^2^. Children start drawing what we call scribbles around their first year of age^3^. At the age of 3–4 years, children begin to produce figurative drawings, i.e. they can be recognized by external observers^3^. This progress is partly linked to the improvement of motor coordination, completed by the progressive integration of a visual vocabulary of patterns and graphic syntax aided by the child’s cultural and living environment and of course by the maturation of their cognitive skills^4,5^. During early childhood, crucial neurological transformations occur in cerebral tissues such as important increases in grey matter volumes until 4 years^6^. This organic substratum allows children for example to acquire the understanding of self and others, to regulate their emotions, but also to communicate and to enter a world filled with symbols^7^. Progressively the systems involved in the drawing behavior develop capacities such as visual perception, graphic production including action programming and planning, and visual imagery. In drawing, symbolism (i.e. the use of symbols such as marks or particular shapes) helps the subject to represent their thoughts by giving them symbolic meaning beyond their literal ones. Then DeLoache and collaborators^8^ stated “the realization of figurative drawing goes hand in hand with the appropriation of symbols”. This early developmental stage of drawing is composed by traces and lines which have a universal nature, belonging to a culturally inherited symbolic system^9^.

One of the first steps of children’s early symbolic drawing development is tadpole figures^10^. Consisting of two lines (legs) attached to a round form (head), the tadpole figure arises around 3 years of age^11^ and is observed in Western as well in non-Western countries^12,13^. Thus, in the same way that children reorganize their spoken language to communicate efficiently, the appearance of these first recognizable drawings also allows them to be better understood by others^14^. An adult can easily understand what a 4-year-old child wanted to represent when they drew, because the outlined objects are recognizable. But what happens if this is not the case? Does this mean that drawings of very young children do not represent anything in particular?

For decades, researchers have suggested that the early phase of drawing—called scribbling—simply reflects a simple motor activity that is visually unplanned and only determined by the motor system of the arm, the wrist and the hand^15^. This would mean that very young children do not take pleasure in their finished drawings, but only in the act itself. Other studies acknowledge that the development of drawing follows a linear trajectory towards realism and that a child’s spontaneous fortuitous realism eventually evolves into representative drawings^16^. However, more recent studies tend to show evidence of pre-representative activities during scribbling^14,17^.

Drawing is part of the larger activity of creating meaning in young children. Children have multiple ways of making sense through the modes, means and materials they use to do so. In their drawings, children encode a lot of information making them a way of constructing and depicting their thoughts in action^18,19^. A 2- or 3-year-old child is not capable of attributing meanings to the entirety of their drawing, which is often unstructured and complex. However, the child can attribute meaning to some parts of their drawing, and especially broken lines, which more easily describe the contours of an object than curved ones^17^. Although caution should be used here because children can change their answers when asked several times about the meaning of their drawings, these kinematic aspects could be considered as precursors of a graphical representation (i.e., the act of producing in visible form a figure or an idea we have in mind). According to Willats^20^, the developmental stages of drawing reflect attempts to represent the child’s ever-increasing levels of perceptual understanding of the world in which they live. In this case, lines drawn in early childhood describe entire objects and are not simply random productions. These suggestions could be of use to answer the continuing debate over the representativeness of drawings by young scribblers. Although the loops, lines and stipples of a scribble usually have no sense for an adult, can we presume that it has no meaning for the young child? Like the young Saint-Exupéry, who complained that adults did not understand his drawing of an elephant eaten by a snake and that “they always need to have things explained”, Longobardi^14^ argues that the inability to detect representativeness in children’s scribbles may be explained by the limitation of adults’ interpretations rather than the absence of this intentionality. In this case, the representativeness of a drawing, namely its concrete and figurative character, would result from the perspectives of two individuals: the entity who produces it (here, a child) and an outside observer who evaluates it (here, an adult). These individuals could be considered to represent two elements: internal representativeness (for the individual who draws) and external representativeness (for the observer). All the aforementioned elements underline the necessity for further studies on drawing behavior to better understand the development of drawing abilities and more specifically the emergence of representativeness in human beings.

An additional factor to consider when seeking to understand the development of drawing behavior in humans may be its evolutionary emergence. Is drawing a typically human behavior or does it originate from ancestor species? This question can be answered by studying the evolution of drawing behavior in species that are genetically close to humans, such as great apes. So far, no spontaneous drawing behavior has been reported in great apes in the wild. Even known as the most meticulous tool makers and users^21^, no chimpanzee was ever observed using sticks to trace on the floor. Moreover, such marking behavior would be difficult to differentiate from other actions such as digging or exploring surfaces. Nevertheless, it is more and more common for captive individuals to use pencils and brushes on paper sheets or even draw on tactile tablets^22^. Indeed, as Call^23^ stated “testing non-human animals outside their ‘natural’ box is needed to fully probe their capabilities and limitations, something that is particularly desirable if our ultimate goal is to reconstruct the evolution of cognition” and in our case, the evolution of drawing. Drawing by chimpanzees has been considered in human-ape comparative developmental studies^24^, and also in art and aesthetics research^25^. In the first experimental study on drawing in chimpanzees, Schiller^26^ presented geometric figures to a female chimpanzee named Alpha who changed her scribbling patterns according to the stimuli provided. A number of studies have shown that chimpanzees maintain their graphic activity without any reinforcement, indicating a likely interest in drawing^25–27^. Beyond the sensation linked to locomotor movement, visual feedback seems to play a reinforcement role: drawing behavior decreases when the line drawn by the subject on the tactile screen disappears^28^. Although there are many studies on mark-making in chimpanzees, none reported drawings that were recognizable by observers (external representativeness), and researchers generally compared the productions to the scribbles of young human children. However, some results such as the trend to change scribbling patterns in presence of stimuli or patterns^5,26^ or, according to Zeller^29^, the manifestation of a choice for some features such as the colors used or patterns drawn leads to conclude that ape drawings “are definitely not random scribbles”.

The most common way to establish whether a drawing is representative or not is to ask the individual who produced it about its meaning, which is often done with children. A sign-language trained female chimpanzee named Moja was asked this question, and she answered “bird”^30^. Of course, her answer is not reliable evidence of her internal representativeness of a bird; it is possible that she answered randomly, or was influenced or misinterpreted by the experimenters. Thus, despite numerous studies on this topic, it is still impossible to exclude the presence of goal-oriented behavior in drawing by very young children and chimpanzees. The use of a symbol requires understanding it, its abstract relation to what it stands for and being able to mentally represent it^8^. In humans, the basic understanding of the analogical space–object–symbol relation emerges around 3 years of age^31^. At this age, a child can understand for example that the illustration of a cat asleep on a couch represents the cat currently sleeping on the living room couch. Besides, pretend play, a symbolic play where children express their imagination by using actions or objects to represent other objects or actions begins in toddlers of around 1–2 years old^32,33^. This capacity of pretense, which is evidence of representational abilities, has also been shown in chimpanzees even if it is less developed^32^. Other abilities, as the fact that they can track invisible displacement^34^ and use a scale model as a source of information for the location of a hidden item^35^ prove that they are capable of mental representation. Then, although their mark-making is not, a priori, driven by a desire to represent an object, the absence of figurative drawings does not necessarily indicate an absence of symbolism for the chimpanzee or the young child^36,37^. Especially as in our sapiens ancestors, “the elaboration of nonfigurative patterns certainly participated in the development of a symbolic thought on which later prospered the invention of the figure and of a true iconographic language”^38^.

Different methods have been used to investigate drawings of young children. The most well-known of these is the comparison of broken lines and curved lines to evaluate the kinematic aspects of the outlines that have been drawn^17^. Although relevant, this method remains subjective as researchers directly question children about what their drawings represent. It cannot therefore be used with very young children (toddlers) or with great apes, as neither group are able to express themselves about their drawings. It is therefore necessary to find a new method of analysis for the objective study of drawing behavior in children and chimpanzees.

To achieve this goal, we asked children aged 3–10 years and adults with different levels of drawing skills (naive versus expert) to draw on tactile devices. Each participant was given two drawing tasks, namely to draw freely (*free* drawing condition) and to draw themselves (*self-portrait* condition) to assess a possible difference in results between a non-specific and a specific task. Five female chimpanzees were also asked to draw freely on a touchscreen tablet. We then developed an innovative and objective index based on the lines drawn by both humans and chimpanzees. This was achieved through the use of spatial analysis. Also called the random walk analysis^39^, this approach is commonly used in ecology to study the movements of animals. We considered the outline of the drawing as an animal’s path (meaning a set of trajectories of different lengths^40^), and characterized the efficiency of the drawing, defined here as the correct reading of the drawing with a minimum of details. In other words, the external representativeness of the observer matches the internal representativeness of the individual who drew. The random walk analysis determines whether the distribution of drawing lines follows a power law or an exponential law. If the distribution follows an exponential law, we expect the drawing to be random, meaning that the individual who is drawing has no intention to represent anything. Contrarily, a power distribution should reflect a non-random and oriented behavior, as found for the daily paths of animals in their natural environments (i.e. goal-oriented and efficient movements^41^). On one hand, we could expect drawing to be random in chimpanzees (i.e. no internal representativeness) since no chimpanzee has ever produced a representative drawing—with a human eye—despite a demonstrated interest in the activity in several studies^25–27^. On the other hand, as chimpanzees are able to change their scribbling outlines and to manifest a preference for colors used or patterns drawn, their drawings might be not so random. Considering humans, we definitely expected a non-random and goal-oriented behavior in children and adults, with an index that increased with age.

Finally, we complemented this innovative spatial index for drawing by investigating the use of colors by individuals and the duration of drawing, since these indices are commonly used in drawing studies. We expected a less developed use of colors in chimpanzees and progressively a more important one with age in humans. Considering the duration of drawing in humans, we predicted a longer one with age in parallel with the improvement of motor and psychic abilities and the growing interest with age for this activity. To study and apprehend the variability of these drawing indices, the variables group (age), gender, test condition, gender-condition interaction and/or the group-condition interaction were used. The gender effect has already been proved to influence drawing in different ways^42–44^. We tested the gender-condition interaction since we thought that, especially for children, the drawing’s variables could be different according condition between girls and boys. Concerning the group-condition interaction, we first expected that the instruction might intimidate some children, especially the youngest, and potentially restrict their creative process. Then experts might react differently from naive adults by being more comfortable and inspired, especially for free drawing, resulting in a potentially longer drawing time and greater use of color.

## Results

### The spatial index µ_MLE_

**Figure 1:**
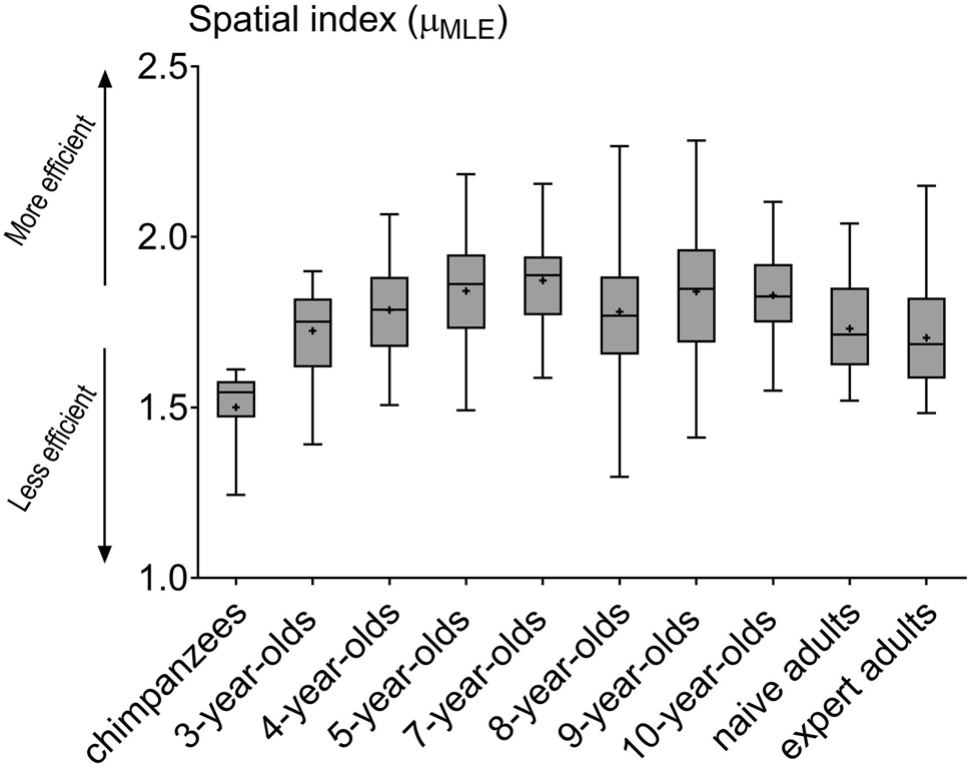
Boxplots of the spatial index μ_MLE_ for each group, i.e. chimpanzees and humans (children and adults). Since the *condition* factor is not present in the selected model, all drawings were studied without distinction of conditions. Each boxplot depicts the median (bold bar), 25–75% quartiles (box), mean (cross) and outliers (points).

Chimpanzees showed a lower index µ_MLE_ than human participants (GLM Gaussian, p < 0.0001, t > 4.10, Fig. 1, Supplementary Table S1A). The index for 3-year-old children was lower than those recorded for 5- (p < 0.001, t = 3.43, Fig. 1, Supplementary Table S1B), 7- (p < 0.0001, t = 4.33), 9- (p < 0.001, t = 3.37) and 10-year-old children (p = 0.002, t = 3.05), but was similar to those of 4- and 8-year-old children and naive or expert adults. Five-year-old children had a higher spatial index µ_MLE_ than the naive (p = 0.032, t = 3.24) and the expert (p = 0.0016, t = 4.03) adults. Seven-year-old children showed an index higher than naive (p = 0.001, t = 4.14) and expert (p < 0.001, t = 4.93) adults. Similarly, 9-year-old children presented a higher spatial index than naive adults (p = 0.039, t = 3.19) and expert adults (p = 0.0023, t = 3.98). Ten-year-old children also had a higher index than expert (p = 0.008, t = 3.66) adults, and possibly also naive (p = 0.097) adults. No difference was found between the two adult groups, and no significant effects of the *conditions* and *sex* factors were observed.

### Drawing duration

**Figure 2:**
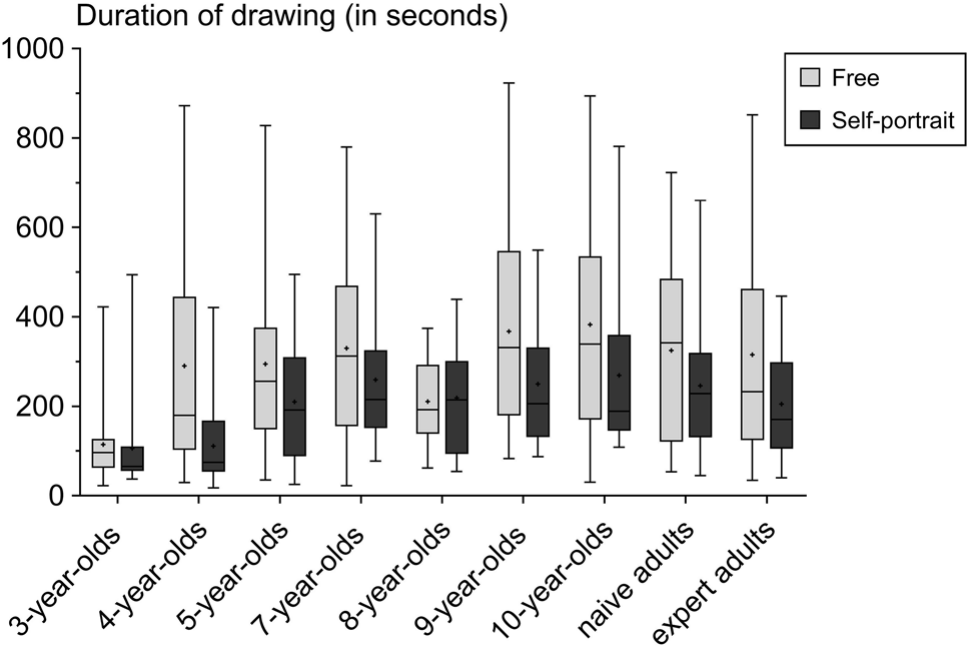
Boxplots of drawing duration (in seconds) for each group and for each condition. Boxplots depict the median (bold bar), 25–75% quartiles (box), mean (cross) and outliers (points).

After model selection, we retained the most explicative model containing the *groups* and *conditions* factors. In both test conditions, 3-year-old children spent less time drawing (mean = 110 ± 97 s) than all other participants (272 ± 192 s) (GLM Gamma, p < 0.001, t > 3.32, Fig. 2, Supplementary Table S2). Furthermore, all human participants spent more time drawing in the *free* condition compared to the *self-portrait* condition (p < 0.0001, t = 4.52).

### The use of colors

#### Number of colors used

**Figure 3:**
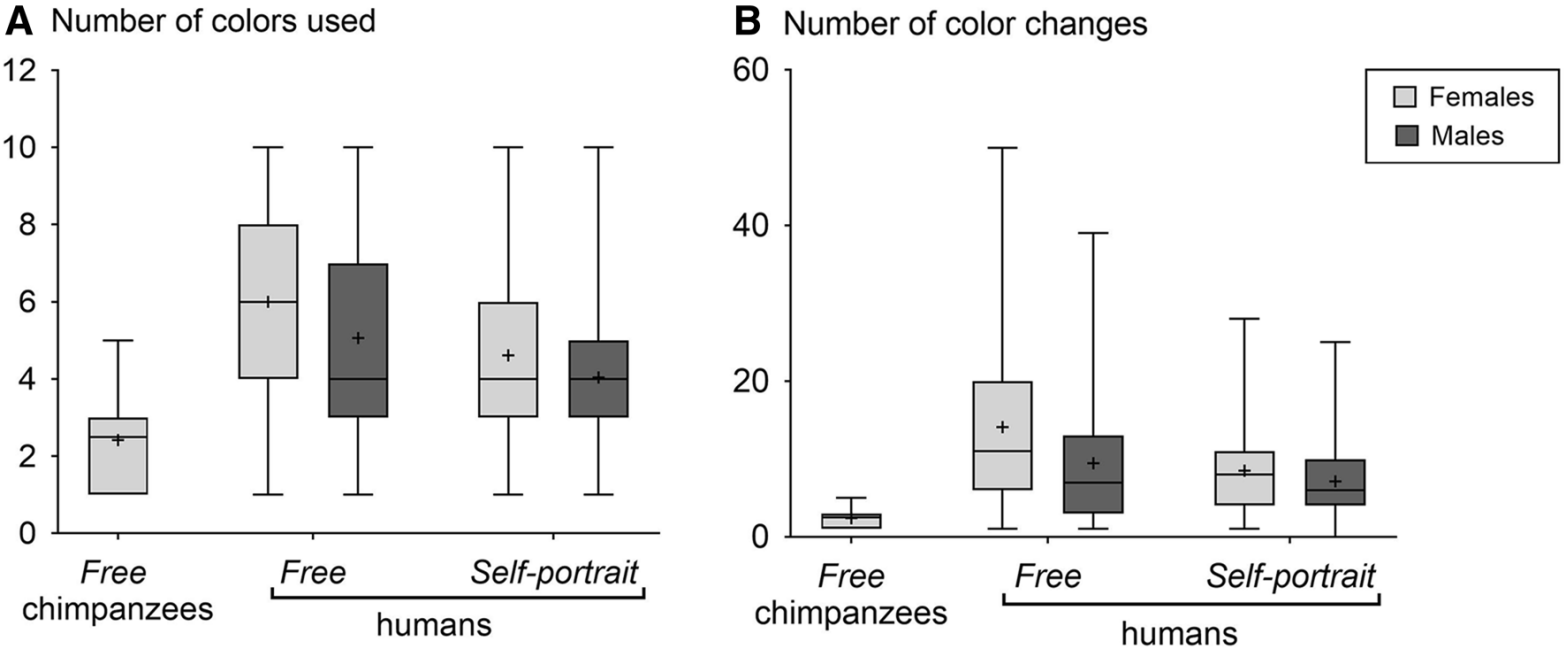
(**A**) Boxplots of the number of colors used by chimpanzees and humans for each condition and for each sex. (**B**) Boxplots of the number of color changes by chimpanzees and humans for each condition and sex. Boxplots depict the median (bold bar), 25–75% quartiles (box), mean (cross) and outliers (points).

The results of the model (Supplementary Table S3A) indicate that chimpanzees used fewer colors than humans (across all groups; GLM Poisson, p ≤ 0.001, t > 3.29, Fig. 3A). After model selection, the factors *conditions* and *sex* were retained to explain the number of colors used in humans. The number of colors used is higher under the *free* condition than under the *self-portrait* condition (p < 0.0001, t = 5.06, Fig. 3A, Supplementary Table S3B). Furthermore, there is a gender-related difference in the use of color. Women and girls used significantly more colors than men and boys (p < 0.001, t = 2.97).

#### Number of color changes

Chimpanzees did not change the colors they used as much as the human participants did (GLM Negative binomial, p < 0.008, t > 3.34, Fig. 3B, Supplementary Table S4A). In humans, the number of color changes was higher in the *free* condition compared to the *self-portrait* condition (GLM Negative binomial, p < 0.0001, t = 4.45, Fig. 3B, Supplementary Table S4B). Furthermore, men and boys changed the colors they used significantly less often than women and girls in all groups (p = 0.002, t = − 3.11).

### The meaning of drawings in children

**Figure 4:**
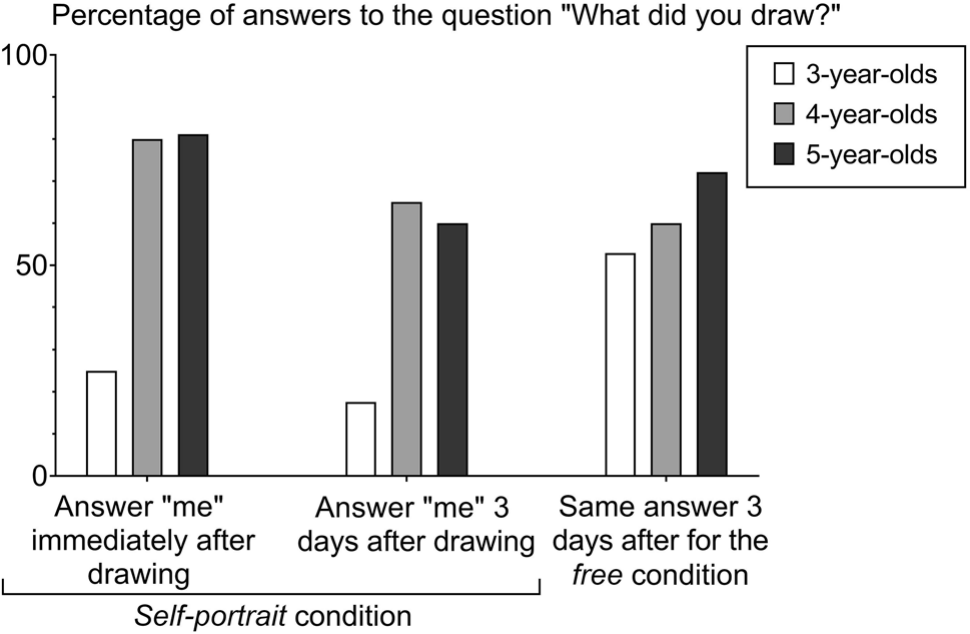
Percentages of answers given by children of 3, 4, 5 years old to the question “What did you draw?” for the *self-portrait* condition and for the *free* condition.

When asked what their drawing represented in the *self-portrait* condition, 3-, 4- and 5-year-old children were much more likely to answer “me” when questioned directly after drawing than when they were asked the same question three days later (GLM Binomial, p < 0.0001, z = 5.16, Fig. 4, Supplementary Table S5). However, these three age groups showed some differences in their response consistency. Four- and five-year-old children were more likely to confirm that the drawing was self-representation immediately after drawing and few days later than the youngest children (p < 0.0001, z > 7.02, Fig. 4, Supplementary Table S5).

The comparison of the answers given by children several days after drawing in the *two conditions* (free and self-portrait) showed that 4- and 5-year-olds had a better memory of what they had intended to represent a few days after drawing than the youngest children (p ≤ 0.021, z > 2.30, Fig. 4, Supplementary Table S6). Three days after the drawing activity, the 3-year-old children seemed to find it easier to remember what they had drawn in the *free* drawing condition (52.9%) than to remember what they had intended to draw or indeed the initial instruction to draw themselves in the *self-portrait* condition (17.6%) (Fig. 4).

## Discussion

This comparative and explorative study allowed us to define new and objective indices characterizing drawing in human and non-human primates. We used common measures in drawing studies (duration and colors) combined with an innovative spatial index that is commonly used in animal trajectories studies^45^. This is the first study to use this mathematical index to understand drawings.

This spatial index μ_MLE_ is clearly lower for chimpanzees compared to human participants. This suggests the presence of a behavior that is less goal-oriented than that observed in humans, but which is still not random: even chimpanzees displayed a power-like distribution showing that their movements are constrained by cognitive or locomotor aspects that limit the randomness we could expect. However, these results conduct us not to conclude to the absence of internal representativeness in chimpanzees. This lack of efficiency is not surprising given their behavior when facing the touchscreen: most of them showed less interest than humans did, as proved by the fact that they were stimulated by the experimenters and their more limited use of colors. This is a surprising contrast to Zeller’s study^29^ which shows that chimpanzees and humans make similar use of colors in their pictures. This difference could be due to the use of the touchscreen, which does not require individuals to use materials such as pencils or paint. Only one chimpanzee, Hatsuka, appeared to be more attentive during test sessions, looking at her fingers and at the outlines produced on the screen. This variability among chimpanzees has already been described, with younger chimpanzees showing more interest^28^. However, Hatsuka’s drawings were not characterized by a higher spatial index than those of other females. This inter-individual difference can be interpreted as a difference of interest in the tool used (i.e. motivation) but did not reveal different line features (i.e. goal-directedness). All chimpanzees produced more or less the same type of very angular and locomotive features with no precise forms. However, these features are not random and it is difficult to conclude to the absence of internal representation in chimpanzees^22^. This question, which has great fundamental implications, remains to be clarified through further experiments.

Among humans, results found were different from those expected. The spatial index μ_MLE_ increased among the two youngest age groups in children and stabilized between the ages of 5 and 10. The spatial index μ_MLE_ then decreased among adults to reach a similar level to that of 3- and 4-year-old children. The increase of this index in young children can be easily explained by the progressive development of more controlled and goal-oriented lines which often underlie the production of a figurative drawing. Goal-directedness and internal representation might be present in some children (at least in the 4-year-olds, where external representation is also present). Three-year-old children would be more motivated by motor pleasure alone^46^, explaining why their spatial index μ_MLE_ is the lowest observed in children. The marks produced on the tablet provided a visual satisfaction which encouraged the child to keep drawing on the blank parts of the screen. However, the index μMLE of 3-year-old children was higher than that observed for chimpanzees, allowing us to develop a hypothesis. First, there was a wide range of variability at this age because it is a period of learning how to draw, in which children begin to internalize some graphic elements (vertical lines, horizontal lines, crosses, dots) that they may or may not be able to reproduce efficiently in their drawings. Many of the children appeared to be in the first representation phase, called action representation, during which the child produces sounds that mimic the movement of the object they want to represent^17,47^, while others began to draw their first recognizable figures. Also, the better fine motor skills of children compared to chimpanzees can be argued. Indeed, even if 3-year-old children did not intend to represent something, the shapes and outlines that they produced (curved or directed to fill the entire available space) did not look like those realized by chimpanzees (which were less smooth). It demonstrates better mobility and control of the hand, the wrist and fingers^48^ on the part of children, which could naturally lead to greater efficiency (e.g. more variation in the length of the lines drawn). The spatial index μ_MLE_ increased in children up to the age of five, when children are in the intellectual realism phase^16^ and try to draw their idea as accurately as possible. This is made possible by the enhancement of their locomotor capacities. The child draws all the parts of the object they seek to depict in an efficient way, e.g. without abstraction or unnecessary details. Here, the primary goal is to be understood (external representativeness), with no aesthetic goal. This makes their drawings more efficient. Among adults, the spatial index μ_MLE_ decreased. Even if their drawings are very understandable, they appear to be more complex due to the compiling of numerous details, perspectives or shadows affecting the distribution of line lengths and consequently reducing their efficiency. Contrary to young children, adults may attempt to reach an ideal in their representations linked to social norms that young children have not yet acquired^49^. The results concerning adults allow a better understanding and definition of our spatial index μ_MLE_. The presence of goal-oriented behavior during drawing is undeniable in adults, almost all of whom produced figurative drawings. They all have an internal representation of their drawings. However, unlike 3-year-old children, the absence of external representation in adult drawings is due to an intention to draw in an abstract way (often observed in the expert group) rather than the absence of internal representativeness. Our spatial index therefore depicts the efficiency of a drawing layout, whether or not it is figurative. It should be noted that the spatial index μ_MLE_ is not affected by the gender of the individual or the condition in which a participant carried out their drawing. Adding an instruction (free or self-portrait condition) to guide the person drawing did not make the drawing layout more efficient.

Even if it does not affect our spatial index, condition has an effect on other measures such as the duration of drawing. Among humans, all groups spent more time drawing under the *free* condition than under the *self-portrait* condition. When free to draw, the participant was willingly using their imagination, and spending more time on their drawing. However, we noted a shorter drawing time for the youngest children, whatever the condition. Three-year-old children appeared to become quickly bored; their use of the tablet was different to that of their older counterparts, who clearly used it as a drawing support. The role of the tablet as medium and the use of fingers as tools could influence several components of the drawing including its efficiency^42^. Finger drawing seems to require less motor control than tools drawing, making it easier for the youngest children to produce more codable, efficient drawings, at least when they copy simple shapes^42^. However, finger drawing may be more difficult for older children and adults, who are more accustomed to draw with brushes or pencils resulting in less qualitative drawings. Holding and moving a tool does not require the same control (distal joints and flexion/extension of the fingers) than drawing with fingers which involves movements of the elbow and the shoulder^50^. It could be more difficult for older children and adults to shift from distal to proximal control of their movements^50^ which could then impact their drawings’ efficiency.

While drawing is generally accepted as the expression of a mental representation, the use of colors is commonly linked to personal aesthetics and is more difficult to evaluate objectively^46^. Although colors alone cannot characterize a drawing, they could help to understand the approach taken by the individual who is drawing. Children and adults used more colors and changed them more often under the *free* condition than in the *self-portrait* condition. The less limited nature of free productions increased the use of colors, while the instruction “*Draw yourself*” seemed to constrain color use and led individuals to use fewer elements when composing the drawing. Besides, the use of colors is consistent with the duration of drawing which is higher under the *free* condition. A gender difference was found for both the number of colors used and the number of colors changes. Women and girls changed the colors they used more frequently and used significantly more colors than men and boys. This gender effect on the number of colors used has already been found in humans, with girls showing a more diverse use of color than boys^44,51^. Gender differences in the use of colors in general have also been shown in studies of drawing by non-human primates^29^. Turgeon^43^ showed a gender difference in color use solely in older children (7–9 years old), whereas we found a difference even in younger children, a finding that is consistent with previous studies^44^. Turgeon^43^ noted that the number of colors used can be directly linked to the subject of the drawing, with some requiring more colors than others (e.g. a flower versus a car). In our case, the gender effect appears in both the constrained (self-portrait) and the free conditions. This gender difference in the use of colors has already been studied, notably from a biological perspective by studying the level of prenatal androgen exposure, even if minimal support was found to assess its role^43^. The importance of social influence is very often highlighted in descriptions of a ‘sex role socialization’ process^44^. Our study shows that this gender difference persists beyond childhood into adulthood.

To complement the analyses of our different indices, we asked the youngest children (between 3 and 5 years of age) about the meaning of their drawings (i.e., internal representativeness) in *free* and *self-portrait* conditions. We asked the children not only at the end of the drawing session but also few days later. Remembering and implementing instructions require the storage and the manipulation of information. For the younger ones for who this exercise could be difficult, we did not hesitate to recall the drawing instruction “*do you remember, I ask you to draw yourself*”. We naturally repeated it to children who looked at the experimenter, asked her to repeat or told her they did not know what to draw. We did not, or very rarely, have to repeat for the oldest children. The fact that we have to recall the instruction could also be a sign of a lack of concentration on the part of the child. For the self-portrait condition, approximately 80% of the 4- and 5-year-old children answered “*me*” immediately after having drawn, identifying themselves in their drawing. This contrasts with the 3-year-old children, who answered “me” significantly less frequently than the older children. Some answered “*nothing*” or “*I do not know*” which may confirm, as our indices do, the absence of an internal representation process for most children of this age. At this age, children are able to form a mental image of themselves^15^, so a misunderstanding of the instruction is not a plausible explanation for our results. On this basis, several hypotheses can be considered. It is possible that 3-year-old children perhaps did not apply our instruction due to a lack of motivation. Also, some of them could have struggled with keeping the instruction in mind during the drawing session. Another hypothesis is that they were simply not capable of carrying out the task since they had not yet integrated the graphical elements necessary for the production of a tadpole man due to developing motor and/or cognitive abilities. We can also consider that despite the habituation phase, some of them were too shy to answer when we asked them. Many 3-year-old children told the experimenter that they “*did not know how to do it*”. This answer can be understood in two ways which are most probably linked: (1) under-developed locomotor control or (2) a partial or incomplete internalization of the graphic elements necessary to draw the figure of a man (round, vertical and horizontal lines). A final hypothesis is that 3-year-old children realized that their drawing did not look like them and therefore could not answer “*me*” when they saw it finished. Among the few 3-year-olds who managed to draw something figurative (i.e., external representativeness), several responded “*a man*”, showing that they did not recognize themselves, which is characteristic of the early period of figurative drawing^52^. It should be noted that the condition in which the drawing was made (free and self-portrait) did not affect the child’s memory of its initial meaning. Like for the self-portrait condition, 3-year-old children did not remember the meaning they had given to their free drawings as well as the oldest children did. In addition to our indices, these results tend to show that the internal representativeness may not well be elaborated in the 3-year-old children, affecting their outlines’ efficiency and therefore the external representativeness of their drawings.

Our study uses an innovative index in this domain and reveals differences between chimpanzees and human beings in their capacities to draw, and further differences between humans at different ages. It is important to note that humans learn to draw—a main activity in nursery school—whilst chimpanzees do not. This can explain some of the differences observed between human and non-human primates. From an early age, a human child raised in an industrial country lives in a graphic environment (books, television, and advertising) that might contribute to the emergence of their figurative conception of drawing^46^. Following this idea, it could be interesting in the future to conduct cross-cultural studies with children living in a less graphic environment. However, one difficulty here would be testing children with technology they are not used to. Chimpanzees can be taught to draw^53–55^ and adults of this species show better control of their movements than younger ones^5^. Then, like in humans, the fine motor skills of chimpanzees improve with age and training, suggesting that the lack of external representativeness in this species is not explained solely by a lack of adequate motor skills. The meticulous examination of productions made by trained chimpanzees over several years could then be an important next step for future studies. Our spatial index μ_MLE_ already reveals the high reliability of efficiency for the lines traced on screens by individuals. The results presented here are preliminary but already show the relevance of pursuing research on new graphical representation clues. New indices should now be developed to understand the degree of representativeness in drawings by primates, and possibly in other animal taxa.

## Methods

### Ethics

Drawings by human participants were confidentially collected. Study protocol followed the ethical guidelines of our research institutions and ethical approval was obtained from the Strasbourg University Research Ethics Committee (Unistra/CER/2019-11). Informed consent was obtained from all adult participants and from a parent or legal guardian for children. Informed consent for publication of identifying images in an online open-access publication has been obtained too.

All chimpanzees were tested in a dedicated testing room and their participation in this study was voluntary^56^. Regular feeding, daily enrichment and ad libitum access to water, leaves, and grasses of live plants were provided^57^. Animal husbandry and research methods complied with international standards (Weatherall report “The use of non-human primates in research”) and all our experimental protocols were approved by Kyoto University (WRC-2017KS009A).

### Subjects

#### Human participants

One hundred and thirty-eight children (63 girls and 75 boys) and forty adults (20 women and 20 men) took part in this study. Children were pupils in a kindergarten and primary school in Strasbourg, France. Twenty children were enrolled for each age group (3-year-old, 4-year-old, 5-year-old, 7-year-old, 8-year-old, 9-year-old and 10-year-old) except for the group of 8-year-olds, which was composed of 18 children (Table 1). Their participation was voluntary and subject to parental consent. Drawings from kindergarten children (3-, 4-, 5-year-olds) were collected in 2018 and drawings from primary school children in 2019. This means that children who were 6 years old in 2019 could not be tested because they have already been involved when they were 5 years old in 2018.

**Table 1:** Groups, sex and test condition of human participants. For each group of participants, half of the subjects began testing with the *free* condition instructions; the other half with the *self-portrait* condition. The choice was made randomly.

Participants groups		Gender	Free = > Self-portrait	Self-portrait = > Free
Children	3-year-olds	Girls	n = 2	n = 3
		Boys	n = 8	n = 7
	4-year-olds	Girls	n = 5	n = 5
		Boys	n = 5	n = 5
	5-year-olds	Girls	n = 5	n = 5
		Boys	n = 5	n = 5
	7-year-olds	Girls	n = 5	n = 5
		Boys	n = 5	n = 5
	8-year-olds	Girls	n = 5	n = 4
		Boys	n = 4	n = 5
	9-year-olds	Girls	n = 5	n = 4
		Boys	n = 5	n = 6
	10-year-olds	Girls	n = 5	n = 5
		Boys	n = 5	n = 5
Adults	Naive	Women	n = 5	n = 5
		Men	n = 5	n = 5
	Expert	Women	n = 5	n = 5
		Men	n = 5	n = 5

The adults tested were 21–60 years old (Table 1). Beyond the age effect (children versus adults), experienced and naive sub-samples were tested in adults to assess the effect of experience on the indices studied. Twenty adults were considered to be naive in drawing since they never took drawing lessons and did not draw as a hobby. These participants were researchers and students of the research institute where the authors worked (naive adults: 30.8 ± 10.54 years-old). Twenty experts in drawing were also enrolled, including art school students and professional illustrators (expert adults: 30.4 ± 11.12 years-old). For both groups, naive and expert, we accepted all ages but made sure to retain the same number of men and women. Participation was voluntary.

#### Chimpanzees

Five female chimpanzees (*Pan troglodytes*) between 10 and 22 years of age were tested at the Kumamoto Sanctuary of the Wildlife Research Center of Kyoto University in Japan (Table 2). Individuals belonged to the same social group of 6 individuals (5 females and 1 male) and lived in a 300 m^2^ enriched and wooded enclosure. All had experienced behavioral and cognitive tests during “participant observation”^56,58^, in which the experimenters interacted directly with the subject during test sessions and were present in the daily lives of the chimpanzees. Chimpanzees had already been familiarized with touchscreens in previous experimental procedures.

**Table 2:** Name and year of birth for each chimpanzee and the number of drawings completed for each test session.

Name	Born in	Session 1	Number of drawings	Session 2	Number of drawings
Natsuki	2005	Tested alone	1	Tested alone	1
Mizuki	1996	Tested with her daughter Iroha	2	Tested with her daughter Iroha	1
Hatsuka	2008	Tested with her mother Misaki	2	Tested with her mother Misaki	2
Misaki	1999	Tested with her daughter Hatsuka	1	Tested with her daughter Hatsuka	1
Iroha	2008	Tested with her mother Mizuki	0	Tested with her mother Mizuki	1

### Experimental design

#### Human participants

**Figure 5:**
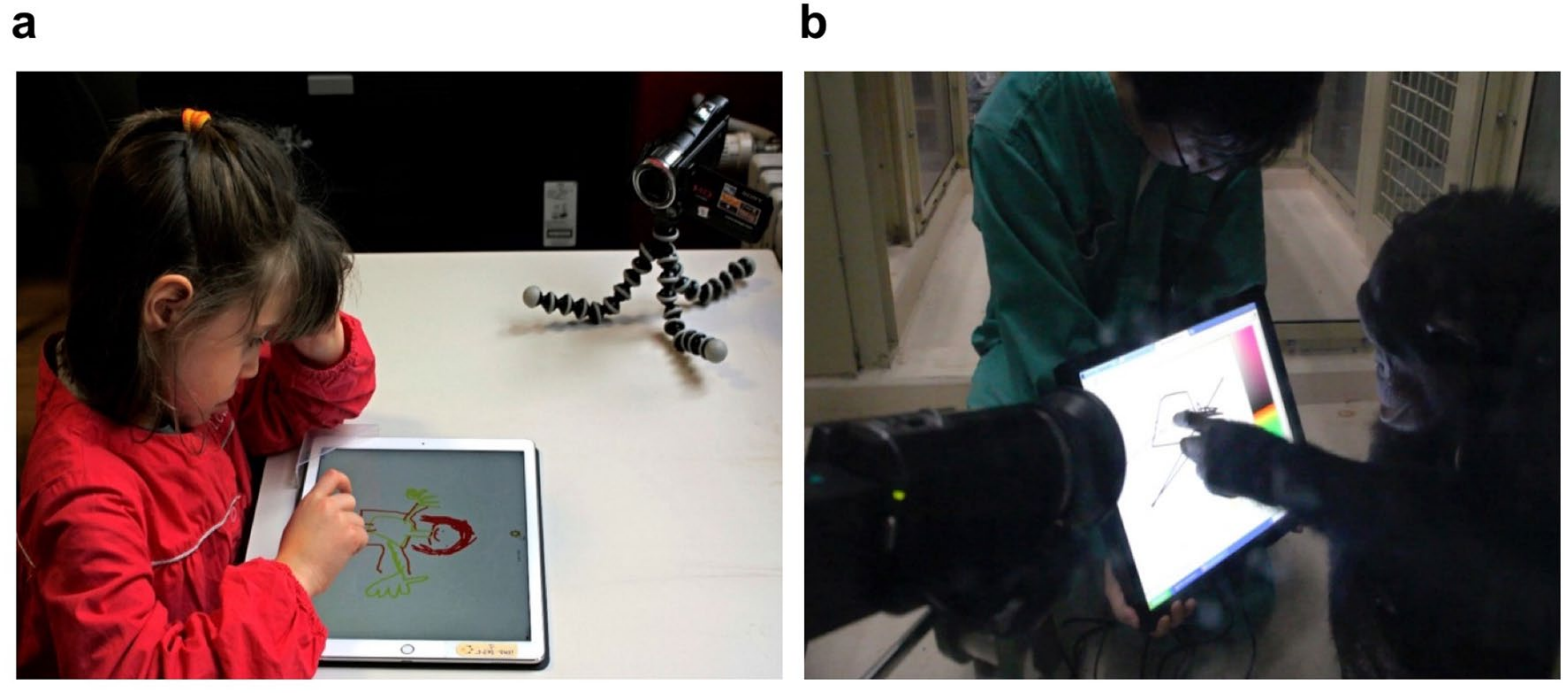
Procedure set up for (**a**) human participants and (**b**) chimpanzees. All drew with their fingers and were filmed during the drawing sessions.

Habituation phase: each participant (children and adults) was invited to try a touchscreen tablet (iPad Pro, 13-Inch, version 11.2.2, capacitive screen reacting to the conductive touch of human fingers), then draw on it with their fingers and understand how it worked, notably to change the color used (Fig. 5a, Supplementary Video S7). The drawing with fingers was preferred to allow the inclusion of some chimpanzees and youngest children who have not yet mastered the use of a pencil. A panel consisting of 10 different colors was displayed on the bottom of the screen, and the participant could select one color for their drawing by clicking on one of them. When they clicked on a different color in the panel, any subsequent drawing production was in that color. Children were habituated the day before the tests to avoid overstimulation. Adults were tested immediately after discovering the tablet.

Testing phase: each child was individually tested at school, during school time, in their classroom for 3-year-olds, and in the staff room for the older children. The experimenter (LM or MP) stayed during the test but kept their distance during drawing in order to avoid influencing the child. Adults were also tested individually in a room at the research institute for naive participants or at the art school for the experts. Contrary to children, adult participants were left alone in the room. A camera recorded the hand movements of all participants while drawing, in case we needed to control for any problem during the session (interruption of the drawing, involuntary tracings, etc*.*). No time limit was applied.

Each participant (child and adult) was tested in two conditions. This choice of drawing tasks was made in order to assess a possible difference in results between a non-specific task (*free* drawing) and a specific one (*self-portrait*).

*Free condition: “Draw what you want”* The experimenter explained to the subject that they could draw whatever they wanted, with no further instructions. The experimenter systematically asked each child up to and including the age of five to say what they had drawn when they had finished their drawing (in older children, it was always obvious what has been drawn). The same question was asked three days later to monitor the consistency of the answer.

*Self-portrait condition: “Draw yourself”* The experimenter instructed the subject to draw themselves. Again, the experimenter systematically asked each child up to and including the age of five what they had drawn, and repeated the question three days later to monitor the consistency of the answer.

In each participant group, half of the subjects began the test with the *free condition* instructions, the other half started with the *self-portrait condition*. The choice was made randomly. To ensure adequate levels of concentration, none of the children drew under both conditions the same day. A total of 356 drawings were collected in humans (Table 1, Supplementary Fig. S8).

#### Chimpanzees

Each female or mother–daughter duo was isolated during lunch time in a testing room using a system of trapdoors and tunnels. Once inside the room, they were provided with fruit and vegetables and asked to sit down. The experimenter (SH) presented a resistive touch screen (1947L 19-Inch Rear-Mount Touch monitor) to the chimpanzee and encouraged her to draw on it using her fingers (Fig. 5b, Supplementary Video S7). Each individual was free to approach and use the touchscreen. There was no time limitation. In the case of mother-daughter duos, some fruit juice was given to the chimpanzee who did not draw to avoid disruptions. The touch screen was connected to a computer that was controlled by a second experimenter (MP), who directly recorded the data for the drawing. Since chimpanzees have a color recognition quite similar to that of humans^59,60^, they also had the opportunity to use colors. A color gradient was displayed on the right-hand side of the screen, and the subject could select a color for her drawing by clicking on it. When she clicked a different color of the panel then any subsequent drawing appeared in that color. Each test session was videotaped in order to analyze the chimpanzee’s behavior when drawing. Chimpanzees drew on two consecutive days in October 2017, and 12 drawings were collected (Table 2**,** Supplementary Fig. S9).

### Statistical analysis

For each drawing, the software allowed us to record the spatial coordinates X and Y of every point of the lines drawn as well as their time coordinates [min; s; ms].

#### The spatial index μ_MLE_

**Figure 6:**
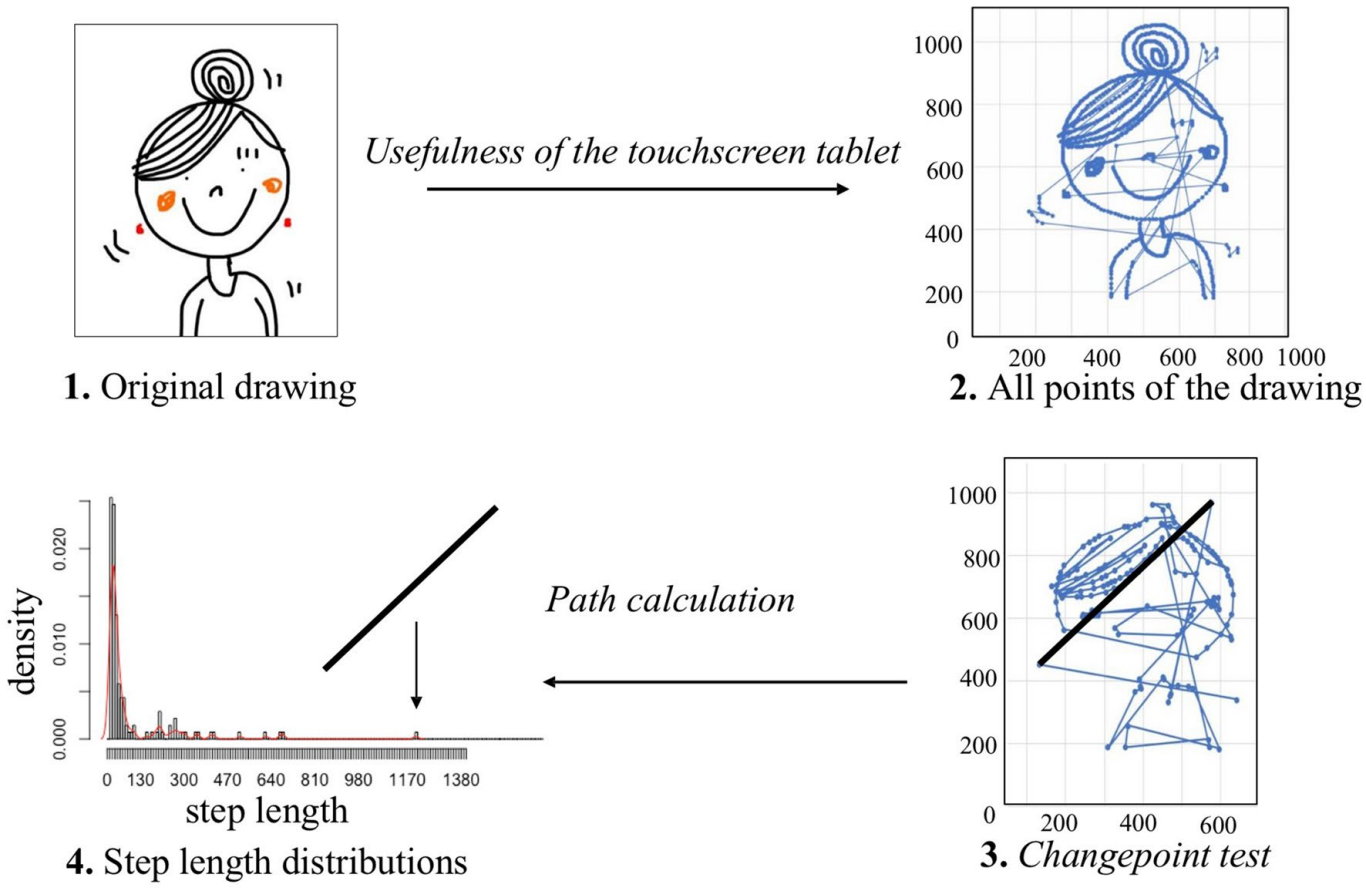
Schema of the different analytical stages for a drawing. All the points of a drawing were extracted from the original one. The application of the changepoint test enabled us to use a reduced number of points; step length distribution was then analyzed. The step length (thick black line) in stage 3 is transferred onto the graph in stage 4. Isabelle Jacqué drew the original drawing.

This first index allowed us to characterize the lines of the drawing. As coordinate scoring of the drawing was continuous (one point per frame), we focused on active changes^41,61^: a selection of points was carried out for each drawing via a change-point test under R software (version 1.1.383; CPT^40^). This allowed us to determine points which included changes of directions and to select solely the active changes of directions produced by the individual who was drawing^40^. This enabled us to limit the number of points considered for each drawing (Fig. 6).

Two consecutive points (i and j) in the drawing *d* determined a step or a vector of a length L(i,j). We then calculated the step lengths S on Excel with latitude x and longitude y (in pixels).

$${\text{S}}\left( {\text{i,j}} \right){ = }\sqrt {{\text{(xj - xi)}}^{{2}} {\text{ + (yj - yi)}}^{{2}} } .$$

Step lengths between 0 and 10 pixels were removed since they often corresponded to very short, inactive movements such as imprecise lines or finger sideslips and caused inaccuracies.

We then determined whether the step length frequency distribution of a drawing followed a power law (y = a × x^μ^) or an exponential law (y = a.e^x × λ^) using the *Maximum Likelihood Method*^45^. To achieve this, we calculated the exponent of the distribution (Eq. 1 for power law and Eq. 2 for exponential law) in order to determine the distribution log-likelihood (Eq. 3 for power law and Eq. 4 for exponential law), where *n* is the total number of step lengths and S_min_ is the minimum step length.

1. Maximum estimate of the power law exponent μ_MLE_:
  $$\mu_{{{\text{MLE}}}} = { 1} + {\text{n }}\left( {\sum {{\text{ln}}\frac{{{\text{Si}}}}{{{\text{Smin}}}}} } \right)^{{ - {1}}}$$
2. Maximum estimate of the exponential law exponent λ_MLE_:
  $$\lambda_{{{\text{MLE}}}} = {\text{ n }}\left( {\sum {\left( {{\text{S}}_{{\text{i}}} - {\text{S}}_{{{\text{min}}}} } \right)} } \right)^{{ - {1}}}$$
3. Log likelihood of the power law L_pow_
  $${\text{L}}_{{{\text{pow}}}} = {\text{n}}({\text{ln}}(\mu_{{{\text{MLE}}}} - {1}) - {\text{ lnS}}_{{{\text{min}}}} ) \, - \mu_{{{\text{MLE}}}} \sum {{\text{lnS}}_{{\text{i}}} /{\text{S}}_{{{\text{min}}}} }$$
4. Log likelihood of the exponential law L_exp_
  $${\text{L}}_{{{\text{exp}}}} = {\text{ nln}}\lambda_{{\text{MLE }}} - \lambda_{{{\text{MLE}}}} \sum {\left( {{\text{S}}_{{\text{i}}} - {\text{S}}_{{{\text{min}}}} } \right)}$$

Log-likelihoods of the exponential and power distributions for each drawing *d* could then be compared using the Akaike Information Criterion (AIC) calculated with L*i,d* the log-likehood of the exponential and of the power distribution and K*i* the number of free parameters *i* in the model (*K* = 1 as the exponent is the only one parameter for both distributions).

$${\text{AIC}}_{{{\text{i}},{\text{d}}}} = \, - {\text{2L}}_{{{\text{i}},{\text{d}}}} + {\text{ 2K}}_{{\text{i}}}$$

The model retained (power or exponential) was that with the lowest AIC, considering a minimum difference of 2 between the two AICs^62^.

All the drawings produced by our 10 groups of participants (chimpanzees, 7 grades of children, naive and expert adults) followed a power law (lowest AIC, see Supplementary Table S10). The Maximum Likelihood Estimate of the power law exponent μ_MLE_ was then used to draw conclusions on the efficiency of the representation for each drawing. This index is comprised of values between 1 and 3^39,63^. The higher the index, the more the line was considered to be directed, well planned and efficient^63^.

#### The drawing duration

In chimpanzees, drawing sessions lasted on average 5 min during which each individual drew less than one minute (mean = 49 ± 17.7 s). However, given that chimpanzees were stimulated by the experimenters and often distracted by their environment, drawing duration was only analyzed in humans. The duration of each drawing was measured in seconds and corresponded to the elapsed time between the first and the last point of the drawing.

#### The use of color

The color of each point has also been recorded by our drawing software. Since the subjects had access to 10 different colors, we defined first a color index ranging from 1 to 10 and corresponding to the number of colors used. We then considered the number of times a subject changed the color used. This second color index can be equal or higher to the number of colors used, as the same color can be used several times.

#### The meaning of drawings in children

When asked about the meaning of their drawing, a child can say what they intended to draw before drawing (internal representativeness) but also what they see once their drawing is finished (fortuitous meaning^16^). If a child remembers the first meaning of their drawing three days later, we can consider this consistency in their answer to be evidence of a real intention to represent the object in question at the outset. This part of the study concerns the three youngest groups of children (between 3 and 5 years old), for whom representative, decipherable and readable drawing (external representativeness) was not systematic.

Each child was questioned immediately after drawing and three days later (LM or MP showed him his drawing again) about the meaning of their drawing in the *self-portrait* and *free* conditions. We chose to not question children before and after their drawing to not disturb them, influence them and their choices, especially the youngest ones who, for some, were initially intimidated by the exercise. Besides, drawing sessions did not last long and children could give the same meaning to their drawing just to match what they had announced, even if plans changed along the way (they did not have the necessary graphic skills to achieve their drawing or at the end, the drawing had no real representational purpose per se). Answers were recorded and coded 1 when the child gave us the same answer at the end of the test session and three days later (for example, “*me*” for the self-portrait condition) and 0 when the child gave us two different answers. First, we analyzed children’s answers for the self-portrait condition. This allowed us to determine whether children were able to understand and follow an instruction, and also indicated their ability to represent something when asked to do so, regardless of whether they could produce a figurative drawing on their own under the free condition. We then compared the answers given by children three days after their drawing in the self-portrait condition with those given three days after drawing in the free condition. This comparison allowed us to determine whether the type of test condition affected the meaning (or the memory thereof) that children had given to their production.

#### Statistics

The multicollinearity of our different indices was tested using a VIF test for each model. As no index had a VIF superior to 4 (VIF_max_ = 2.71), we concluded that there was no collinearity between our variables^64^. This means that the spatial index, the color used and the time taken to draw are not dependent on each other.

The indices were analyzed in two different ways. First, we compared human participants and chimpanzees by taking only the *free* condition drawings into account. The sex factor was excluded as all chimpanzees were female. In a second step, we compared *gender*, *test condition* and *groups* within the groups of human participants*.* No influence of condition order was found (Wilcoxon test, W < 303, p > 0.05207) meaning that data did not significantly differ whether the person first drew under *free* or *self-portrait* conditions.

Each index was studied through Generalized Linear Models GLM (dependent variable ~ explanatory variables). *Individual *(*IDs*) was added as a random factor when considering the meanings of drawings in children. We first selected the most efficient model to explain the variability of the studied index, using the variables group, *gender*, *test condition*, *gender-condition* interaction and/or *group-condition* interaction. The model selection was carried out by a dredge (package MuMin^65^) which is a forward procedure (from the null to complete model). After the dredge, we selected the one with the lowest AIC (model selection^65^). When the AIC difference of the first two best models did not exceed two^62^, they were compared using an Anova [anova function (glm1, glm2, test: “F” or “Chisq”)]. When the difference was not significant (p > 0.05), the simplest model was preferred to respect the parsimony principle. For each selected model, the probability distribution was adapted to the dependent variable and the conditions of application (normality and homoscedasticity of residuals) were graphically verified. All analyses were performed in R. 3.5.0^66^ and for all tests, the significance threshold was set to α = 0.05.

## Supplementary Information

Supplementary Video 1.

Supplementary Information 1.

Supplementary Information 2.

## Data Availability

Information and data are available in the Supplementary Materials, and additional data related to this paper may be requested from the authors.

## References

[1] Martinet, L. & Pelé, M. Sortir du cadre: désanthropiser le concept de dessin en questionnant les primates non humains. In *Pourquoi Désanthropiser et Décloisonner* (eds. Baratay, E.) (Editions de La Sorbonne, in press).

[2] MacDonald J (2014). Alpha: The figure in the cage. Relat. Beyond Anthropocentrism..

[3] Golomb C (1992). The Child’s Creation of a Pictorial World.

[4] Cohn N (2012). Explaining, “I can’t draw”: Parallels between the structure and development of language and drawing. Hum. Dev..

[5] Saito A, Hayashi M, Takeshita H, Matsuzawa T (2014). The origin of representational drawing: A comparison of human children and chimpanzees. Child Dev..

[6] Coulbeau L, Royer P, Brouziyne M, Dosseville F, Molinaro C (2008). Development of children’s mental representations: Effects of age, sex, and school experience. Percept. Motor Skill..

[7] Brownell CA, Kopp CB (2007). Socioemotional Development in the Toddler Years: Transitions and Transformations.

[8] DeLoache, J. S., Pierroutsakos, S. L. & Troseth, G. L. The three 'R's of pictoral competence. In *Annals of Child Development* (eds. Vasta, R.) 1–48 (Jessica Kingsley, Bristol, 1996).

[9] Golomb C (2002). Child Art in Context: A Cultural and Comparative Perspective.

[10] DeLoache JS (2004). Becoming symbol-minded. Trends Cogn. Sci..

[11] Cox MV (1993). Children’s Drawings of the Human Figure.

[12] Gernhardt A, Rübeling H, Keller H (2015). Cultural perspectives on children’s tadpole drawings: At the interface between representation and production. Front. Psychol..

[13] Porte, G. Dessine-toi. http://www.early-pictures.ch/porte1/en/ (2010).

[14] Longobardi C, Quaglia R, Lotti NO (2015). Reconsidering the scribbling stage of drawing: A new perspective on toddlers’ representational processes. Front. Psychol..

[15] Piaget J, Inhelder BL (1966). Psychologie de l’Enfant.

[16] Le Luquet GH (1927). Dessin Enfantin.

[17] Adi-Japha E, Levin I, Solomon S (1998). Emergence of representation in drawing: The relation between kinematics and referential aspects. Cogn. Dev..

[18] Matthews J (1999). The Art of Childhood and Adolescence: The Construction of Meaning.

[19] Kress G (1997). Before Writing: Re-thinking the Paths to Literacy.

[20] Willats J (2005). Making Sense of Children’s Drawings.

[21] McGrew W (1992). Chimpanzee Material Culture: Implications for Human Evolution.

[22] Martinet, L. & Pelé, M. Drawing in non-human primates: What we know and what remains to be investigated. *J. Comp. Psychol.* Available online (2020) 10.1037/com0000251 33252920

[23] Call, J. On space geckos and urban apes. In *Diversity in Harmony: Insights from Psychology-Proceedings of the 31st International Congress of Psychology* 42 (Wiley, Hoboken, 2018).

[24] Kellogg WN, Kellogg LA (1933). The Ape and the Child: A Study of Environmental Influence Upon Early Behaviour.

[25] Morris D (1962). The Biology of Art: A Study of the Picture-making Behavior of the Great Apes and its Relationship to Human Art.

[26] Schiller PH (1951). Figural preferences in the drawings of a chimpanzee. J. Comp. Physiol. Psychol..

[27] Hanazuka Y, Kurotori H, Shimizu M, Midorikawa A (2019). The effects of the environment on the drawings of an extraordinary productive Orangutan (*Pongo pygmaeus*) artist. Front. Psychol..

[28] Tanaka M, Tomonaga M, Matsuzawa T (2003). Finger drawing by infant chimpanzees (*Pan troglodytes)*. Anim. Cogn..

[29] Zeller A (2007). What’s a picture ? A comparison of drawings by apes and children. Semiotica..

[30] Gardner RA, Gardner BT (1978). Comparative psychology and language acquisition. Ann. NY. Acad. Sci..

[31] DeLoache JS (1987). Rapid change in the symbolic functioning of very young children. Science.

[32] Matsuzawa T (2020). Pretense in chimpanzees. Primates.

[33] Fein G (1981). Pretend play in childhood: An integrative review. Child Dev..

[34] Collier-Baker E, Davis JM, Nielsen M, Suddendorf T (2006). Do chimpanzees (*Pan troglodytes*) understand single invisible displacement?. Anim. Cogn..

[35] Kuhlmeier VA, Boysen ST (2002). Chimpanzees (*Pan troglodytes*) recognize spatial and object correspondences between a scale model and its referent. Psychol. Sci..

[36] Savage-Rumbaugh ES, Rumbaugh DM, Boysen S (1978). Symbolic communication between two chimpanzees (*Pan troglodytes*). Science.

[37] DeLoache JS (1991). Symbolic functioning in very young children: Understanding of pictures and models. Child Dev..

[38] Bon, F. Dis, dessine-moi un mammouth. In *Sapiens à l’œil nu* (eds CNRS, 2019).

[39] Bartumeus F, da Luz MGE, Viswanathan GM, Catalan J (2005). Animal search strategies: A quantitative random-walk analysis. Ecology.

[40] Byrne RW, Noser R, Bates LA, Jupp PE (2009). How did they get here from there? Detecting changes of direction in terrestrial ranging. Anim. Behav..

[41] Sueur C, Briard L, Petit O (2011). Individual of Lévy walk in semi-free ranging Tonkean macaques (*Macaca tonkeana*). PLoS ONE.

[42] Kirkorian HL, Travers BG, Jiang MJ, Choi K, Rosengren KS, Pavalko P, Tolkin E (2020). Drawing across media: A cross-sectional experiment on preschoolers’ drawings produced usin traditional versus electronic mediums. Dev. Psychol..

[43] Turgeon SM (2008). Sex differences in children’s free drawings and their relationship to 2D:4D ratio. Pers. Individ. Differ..

[44] Wright L, Black F (2013). Monochrome males and colorful females: Do gender and age influence the color and content of drawings?. SAGE Open..

[45] Edwards AM (2007). Revisiting Lévy flight search patterns of wandering albatrosses, bumblebees and deer. Nature.

[46] Baldy, R. *Fais-moi un Beau Dessin: Regarder le Dessin de l’Enfant, Comprendre son Evolution* (Paris, France: In Press, 2011).

[47] Yamagata K (2001). Emergence of representational activity during the early drawing stage: Process analysis. Jpn. Psychol. Res..

[48] Young RW (2003). Evolution of the human hand: The role of throwing and clubbing. J. Anat..

[49] Itskowitz R, Glaubman H, Hoffman M (1988). The impact of age and artistic inclination on the use of articulation and line quality in similarity and preference judgments. J. Exp. Child Psychol..

[50] Picard D, Martin P, Tsao R (2014). iPads at school? A quantitative comparison of elementary schoolchildren’s pen-on-paper versus finger-on-screen drawing skills. J. Educ. Comput. Res..

[51] Milne LC, Greenway P (1999). Color in children’s drawings: The influence of age and gender. Arts Psychother..

[52] Cox MV, Parkin CE (1986). Young children’s human figure drawing: Cross-sectional and longitudinal studies. Educ. Psychol..

[53] Iversen IH, Matsuzawa T (1996). Visually guided drawing in the chimpanzee (*Pan troglodytes)*. Jpn. Psychol. Res..

[54] Iversen IH, Matsuzawa T (1997). Model-guided line drawing in the chimpanzee (*Pan troglodytes*). Jpn. Psychol. Res..

[55] Iversen, I. H. & Matsuzawa, T. Establishing line tracing on a touch monitor as a basic drawing skill in chimpanzees (*Pan troglodytes*). In *Primate Origins of Human Cognition and Behavior* 235–268 (Springer, Tokyo, 2001).

[56] Matsuzawa T, Tomonaga M, Tanaka M (2006). Cognitive Development in Chimpanzees.

[57] Matsuzawa T (2020). WISH cages: Constructing multiple habitats for captive chimpanzees. Primates.

[58] Matsuzawa T (2017). The 40th anniversary of the Ai Project: The commemorative gift is a silk scarf painted by Ai the chimpanzee. Primates.

[59] Matsuzawa T (1985). Colour naming and classification in a chimpanzee (*Pan troglodytes*). J. Hum. Evol..

[60] Matsuno T, Kawai N, Matsuzawa T (2004). Color classification by chimpanzees (*Pan troglodytes*) in a matching-to-sample task. Behav. Brain Res..

[61] Noser R, Byrne RW (2014). Change point analysis of travel routes reveals novel insights into foraging strategies and cognitive maps of wild baboons. Am. J. Primatol..

[62] Burnham KP, Anderson DR (2004). Multimodel inference: Understanding AIC and BIC in model selection. Sociol. Methods Res..

[63] Viswanathan GM, Buldyrev SV, Havlin S, da Luz MGE, Raposo EP, Stanley HE (1999). Optimizing the success of random searches. Nature.

[64] Dormann CF (2012). Collinearity: A review of methods to deal with it and a simulation study evaluating their performance. Ecography.

[65] Barton, K. MuMIn: multi-model inference, R package version 0.12.0. http://r-forge.r-project.org/projects/mumin/ (2009).

[66] R Core Team *R: A Language and Environment for Statistical Computing* https://www.R-project.org (Vienna, Austria, 2018).

