# Supplementary material for: New indices to characterize drawing behavior in humans (Homo sapiens) and chimpanzees (Pan troglodytes)
Source: Sci Rep. 2021 Feb 16;11:3860. doi: 10.1038/s41598-021-83043-0 (PMC7887262; doi:10.1038/s41598-021-83043-0)
Supplement: Supplementary file 2 — Supplementary Information 1. [file 41598_2021_83043_MOESM2_ESM.pdf]

Supplementary materials for the article "New indices to characterize drawing behavior in humans (*Homo sapiens*) and chimpanzees (*Pan troglodytes*)"

Lison Martinet, Cédric Sueur, Satoshi Hirata, Jérôme Hosselet, Tetsuro Matsuzawa, Marie Pelé

Results

1. The spatial index μ_{MLE}

Table S1. Results of the generalized linear model realized to compare the spatial fractal index μ_{MLE}. **(a)** between chimpanzees and humans (compared to chimpanzees) and **(b)** among humans (compared to 3-year-old children).

		Estimate	Std. Error	t value	Pr(>|t|)
a	Intercept	1.50	0.04	34.91	< 0.0001
	3-year-old children	0.22	0.05	4.51	< 0.0001
	4-year-old children	0.28	0.05	5.73	< 0.0001
	5-year-old children	0.34	0.05	6.85	< 0.0001
	7-year-old children	0.37	0.05	7.47	< 0.0001
	8-year-old children	0.28	0.05	5.55	< 0.0001
	9-year-old children	0.34	0.05	6.82	< 0.0001
	10-year-old children	0.33	0.05	6.60	< 0.0001
	Naive adults	0.23	0.05	4.63	< 0.0001
	Expert adults	0.20	0.05	4.10	< 0.0001
b	Intercept	1.72	0.03	71.72	< 0.0001
	4-year-old children	0.06	0.03	1.79	0.07
	5-year-old children	0.12	0.03	3.43	< 0.001
	7-year-old children	0.14	0.03	4.33	< 0.0001
	8-year-old children	0.05	0.03	1.58	0.11
	9-year-old children	0.11	0.03	3.37	< 0.001
	10-year-old children	0.10	0.03	3.05	0.002
	Naive adults	0.01	0.03	0.19	0.85
	Expert adults	-0.02	0.03	-0.61	0.54

2. The drawing duration

Table S2. Results of the generalized linear model to study drawing duration among humans (compared to 3-year-old children, and *free* condition compared to *self-portrait* condition).

	**Estimate**	**Std. Error**	**t value**	**Pr(>|t|)**
Intercept	4.54	0.12	38.41	< 0.0001
4-year-old children	0.52	0.16	3.32	< 0.001
5-year-old children	0.80	0.16	5.08	< 0.0001
7-year-old children	0.96	0.16	6.11	< 0.0001
8-year-old children	0.67	0.16	4.15	< 0.0001
9-year-old children	1.06	0.16	6.33	< 0.0001
10-year-old children	1.06	0.16	6.69	< 0.0001
Naive adults	0.93	0.16	5.89	< 0.0001
Expert adults	0.83	0.16	5.23	< 0.0001
Free condition	0.33	0.07	4.52	< 0.0001

3. The use of colors

a. Number of colors used

Table S3. Results of the generalized linear model carried out to compare the number of colors used. **(a)** between chimpanzees and humans (compared to chimpanzees) and **(b)** among humans (*free* condition compared to *self-portrait* condition and boys/men compared to girls/women).

		**Estimate**	**Std. Error**	**z value**	**Pr(<|z|)**
**a**	Intercept	0.88	0.18	4.75	< 0.0001
	3-year-old children	0.88	0.21	4.26	< 0.0001
	4-year-old children	1.06	0.20	5.21	< 0.0001
	5-year-old children	0.81	0.21	3.89	< 0.0001
	7-year-old children	0.77	0.21	3.65	< 0.001
	8-year-old children	0.77	0.21	3.63	<0.001
	9-year-old children	0.70	0.21	3.29	< 0.001
	10-year-old children	0.88	0.21	4.26	< 0.0001
	Naive adults	0.71	0.21	3.34	< 0.001
	Expert adults	0.74	0.21	3.60	< 0.001
**b**	Intercept	1.45	0.04	35.56	< 0.0001
	Free condition	0.24	0.05	5.06	< 0.0001
	Boys/men	-0.14	0.05	-2.97	< 0.001

b. Number of color changes

Table S4. Results of the generalized linear model to compare the number of color changes. **(a)** between chimpanzees and humans (compared to chimpanzees) and **(b)** among humans (*free* condition compared to the *self-portrait* condition and boys/men compared to girls/women).

		Estimate	Std. Error	z value	Pr(>|z|)
**a**	Intercept	0.35	0.35	0.98	0.33
	3-year-old children	1.40	0.42	3.34	< 0.001
	4-year-old children	2.14	0.41	5.17	< 0.0001
	5-year-old children	1.99	0.41	4.81	< 0.0001
	7-year-old children	2.02	0.41	4.87	< 0.0001
	8-year-old children	1.92	0.42	4.56	< 0.0001
	9-year-old children	2.02	0.41	4.88	< 0.0001
	10-year-old children	1.95	0.41	4.70	< 0.0001
	Naive adults	2.10	0.41	5.08	< 0.0001
	Expert adults	2.34	0.41	5.71	< 0.0001
**b**	Intercept	2.07	0.09	24.02	< 0.0001
	Boys/men	-0.30	0.1	-3.11	0.002
	Free condition	0.43	0.1	4.45	<0.0001

4. The meaning of drawings in children

Table S5. Results of the generalized linear model carried out to study the consistency of the answer for the *self-portrait* condition among the three youngest categories of children (compared to the 3-year-old children and the response rate « me » immediately compared to « me » after three days).

	Estimate	Std. Error	z value	Pr(>|z|)
Intercept	-26.99	4.48	-6.03	< 0.0001
4 year-old children	39.12	5.57	7.02	< 0.0001
5 year-old children	38.76	5.51	7.04	< 0.0001
Response rate « Me » immediately after the *self-portrait* condition	14.34	2.78	5.16	< 0.0001

Table S6. Results of the generalized linear model carried out to study the effect of the condition on memorization (compared to the 3-year-old children).

	Estimate	Std. Error	z value	Pr(>|z|)
Intercept	-0.61	0.36	-1.69	0.091
4 year-old children	1.12	0.48	2.30	0.021
5 year-old children	1.26	0.49	2.54	0.011

Data collection and analysis

Video file S7. Short sequence of an adult's drawing session on the touchscreen tablet (iPad Pro, 13-Inch, version 11.2.2) followed by a short sequence of a chimpanzee's drawing session on the resistive touchscreen (1947L 19-Inch Rear-Mount Touch monitor).

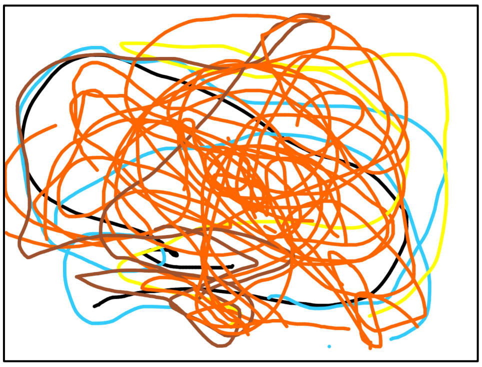

Self-portrait condition

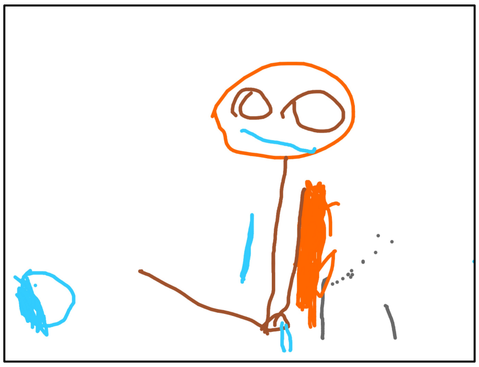

Self-portrait condition

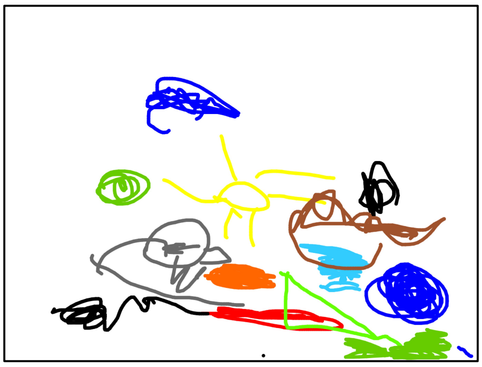

Free condition

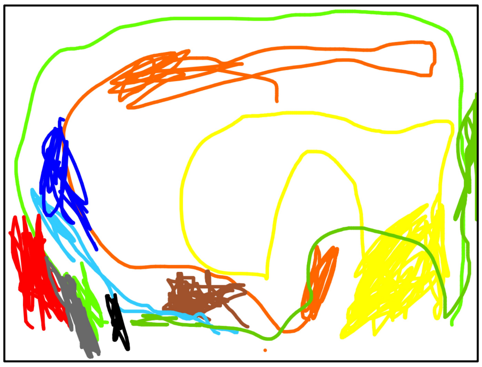

Free condition

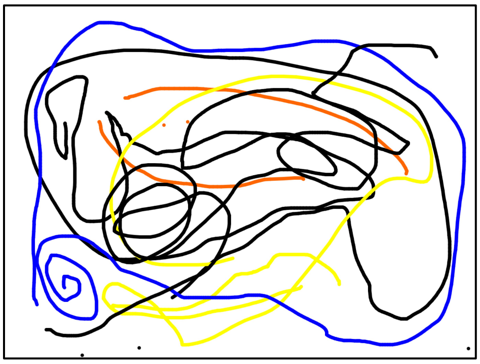

Free condition

Figure S8. Examples of drawings by 3-year-old children, in *free* and *self-portrait* conditions.

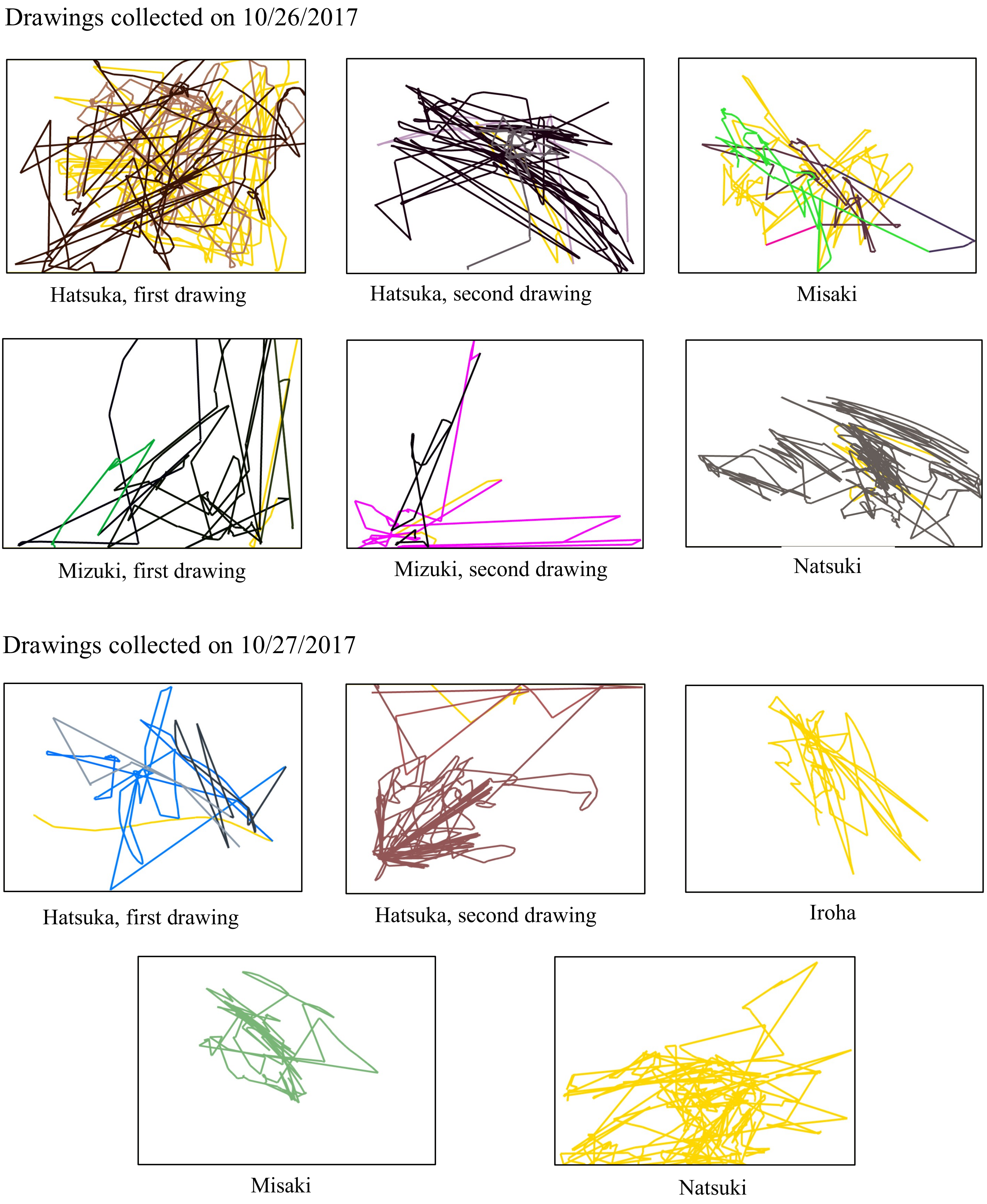

Figure S9. Chimpanzees' drawings collected on two consecutive days in October 2017. During the second day, one of the females (Mizuki) erased her drawing but the data had been saved.

Table S10. Table with data used for the analysis, see "Data table" excel file.
